# Decreased expression of CCL17 in the disrupted nasal polyp epithelium and its regulation by IL-4 and IL-5

**DOI:** 10.1371/journal.pone.0197355

**Published:** 2018-05-10

**Authors:** Byoungjae Kim, Hyun-Ji Lee, Nu-Ri Im, Doh Young Lee, Ha Kyun Kim, Cha Young Kang, Il-Ho Park, Seung Hoon Lee, Heung-Man Lee, Sang Hag Lee, Seung-Kuk Baek, Tae Hoon Kim

**Affiliations:** 1 Department of Otorhinolaryngology-Head & Neck Surgery, College of Medicine, Korea University, Seoul, South Korea; 2 Neuroscience Research Institute, Korea University, College of Medicine, Seoul, Republic of Korea; Mie University Graduate School of Medicine, JAPAN

## Abstract

**Background:**

In airway epithelium, thymus and activation-regulated chemokine (CCL17) and macrophage-derived chemokine (CCL22) are induced by defective epithelial barriers such as E-cadherin and attract the effector cells of Th2 immunity. However, the association between the epithelial barrier and CCL17 expression has not been studied in chronic rhinosinusitis with nasal polyp (CRSwNP). Thus, we aimed to evaluate the expression of CCL17 and its regulation by Th cytokines in nasal polyp (NP) epithelial cells.

**Methods:**

The expression and distribution of CCL17, CCL22, E-cadherin and/or epidermal growth factor receptor (EGFR) were measured using real-time PCR, western blot, and immunohistochemistry and compared between normal ethmoid sinus epithelium and NP epithelium. In addition, the expression level of CCL17 was determined in cultured epithelial cells treated with IL-4, IL-5, IL-13, TNF-α, and IFN-γ.

**Results:**

The expression of CCL17 was decreased in the NP epithelium compared to the epithelium of normal ethmoid sinus, whereas the expression of CCL22 was not decreased. E-cadherin was differentially distributed between the epithelium of normal ethmoid sinus and NP epithelium. EGFR was also decreased in NPs. Interestingly, the stimulation of cultured epithelial cells with Th2 cytokines, IL-4 and IL-5, resulted in an upregulation of CCL17 expression only in NP epithelial cells whereas the expression of CCL17 was increased in both normal epithelial cells and NP epithelial cells by Th1 cytokines.

**Conclusion:**

Our results suggest that the decreased expression of CCL17 in defective NP epithelium may be closely connected to NP pathogenesis and can be differentially regulated by cytokines in the NP epithelium of patients with CRSwNP.

## Introduction

Chronic rhinosinusitis (CRS) is a chronic and persistent inflammatory disease of the sinonasal mucosa. The pathogenesis of CRS remains poorly understood and it is difficult to treat. A nasal polyp (NP) is a mucosal sac containing edema, fibrous tissue, vessels, inflammatory cells, and glands with disrupted epithelium.[1] NPs frequently accompany CRS, and their presence complicates the treatment of this disease.[2] CRS with NP (CRSwNP) in Asian patients predominantly show a mixed T cell immune response whereas CRSwNP in Western patients is typically characterized by Th2 cytokine polarization.[3, 4] Although the mechanisms that induce the Th2 response seem integral to the pathogenesis of CRSwNP, these mechanisms are not fully understood in Asian patients.[5]

Disruption of the airway epithelium with bacteria, viruses, and pollutants causes the initial danger signal that incites cytokine/chemokine secretion from epithelial cells to initiate the Th response.[6] Thymic stromal lymphopoietin (TSLP), an epithelial cell-derived cytokine, plays an important role in the development of the Th2 response in nasal polyps.[7] There remains some discrepancy regarding whether IL-25 and IL-33 levels, other epithelium-derived cytokines/chemokines that promote Th2 inflammation, are increased in NPs.[8–12]

Chemokines are small 8–10 kDa cytokines that induce the migration of various types of leukocytes.[13] The thymus and activation-regulated chemokine (TARC, CCL17) and macrophage-derived chemokine (MDC, CCL22) selectively attract Th2 cells or regulatory T cells, and are therefore considered to play an important role in Th2-type inflammation.[6, 14–16] CCL17 is produced by monocytes, dendritic cells and epithelial cells and its expression increases in asthmatic epithelial cells.[16, 17] Recently, it was reported that a defective epithelial barrier, such as E-cadherin, can induce epithelial CCL17 production and its expression can be regulated by cytokines.[18, 19]

Considering that CCL17 and CCL22 are epithelium-derived chemokines involved in Th2 inflammation of the airway mucosa, altered levels of expression of these chemokines may play an role in the pathogenesis of NPs. However, the precise participation of these chemokines in NPs has not been evaluated. Therefore, the aims of the present study were as follows:

1. To determine the expression levels and distributional patterns of CCL17, CCL22 and E-cadherin using real-time PCR, Western blot, and immunohistochemistry within the ethmoid sinus mucosa of healthy controls and the polyp of patients with CRSwNP to evaluate the possible effects of CCL17 and CCL22 in the pathogenesis of NPs.
2. To elucidate whether the expression of CCL17 is altered upon stimulation with cytokines relevant to CRSwNP.

## Materials and methods

### Tissue preparation

Fifteen patients with CRSwNP and 15 controls were enrolled. Control subjects were patients without sinus disease undergoing endoscopic reduction because of a blowout fracture and didn’t have any sinus disease. Endoscopic physical findings, computed tomographic scoring systems, and SNOT-20 were rated, as described previously.[20–22] Clinical data of patients including physical findings and the CT scoring system are summarized in Table 1.

**Table 1:** Patients characteristics.

	Controls	CRSwNP
No. of patients	15	15
No. (female/male)	5/10	4/11
Age (y), mean (range)	29. 5 (18–35)	37.6 (17–40)
Asthma history	0	0
Skin test result	Negative	Negative
Smoking	2/15	3/15
No. of sinus surgery	0	0
SNOT-20 score*	2.5 ± 1.6	27.9 ± 2.5
CT grade*	0	17.8 ± 2.3
Endoscopy score *	0	8.5 ± 0.9
Total inflammatory cells*	11.7 ± 5.8	77.5 ± 8.8
Eosinophils*	1.0 ± 0.2	4.9 ± 1.5
Mononuclear cells*	15.5 ± 6.9	45.3 ± 12.1
No. of Polyp		15
Eosinophilic		1
Non-eosinophilic		14

*SNOT*-20, 20-item Sino-Nasal Outcome Test.

*The data of CRSwNP are significantly higher than those of controls.

The diagnosis of CRS was made according to the current European position paper on rhinosinusitis and NP.[23] None of the control subjects or patients with CRSwNP had a history of asthma or a positive skin prick test result to a standard panel of aeroallergens. Subjects were excluded if they used any oral or topical medication including steroids, antihistamines, or antibiotics at least 3 months prior to the surgery. The institutional review board for human subjects at the Korea University Medical Center approved the protocols and the informed consent form, and written informed consent was obtained from all patients before collecting any samples for experimental purposes. Normal ethmoid sinus mucosa was obtained intraoperatively from the ethmoid sinus during endoscopic reduction in patients with a blowout fracture. During operation, the normal-appearing ethmoid sinus mucosa, which was not injured by the fracture, was obtained as a normal control. NP tissue was obtained from patients undergoing endoscopic sinus surgery for CRSwNP.

All tissue samples were divided into two sections. The first section was immersed in a fixative containing 4% paraformaldehyde for histology or extracted for real time PCR. Sections stained with hematoxylin and eosin were examined with a light microscope to obtain a general impression of the typical pathologic features encountered in normal sinus mucosa and nasal polyp. The numbers of total inflammatory cells, eosinophils, and mononuclear cells in the subepithelial layer was counted to determine the mean percentage of eosinophils out of the total number of inflammatory cells at high-power magnification (X400), and 5 high-power fields were randomly selected by two independent physicians. Patients with CRSwNP were classified as eosinophilic CRSwNP when more than 10% of the total observed inflammatory cells were eosinophils and as non-eosinophilic CRSwNP otherwise. Fourteen NP tissues out of fifteen patients with CRSwNP were non-eosinophilic CRSwNP. (Table I) The second section was treated with dispase (1 mg/ml, GenDEPOT, Katy, TX, USA) to harvest epithelial cells.

To harvest epithelial cells, tissues were treated with dispase for 24 hours at 4°C. Cells were detached from the enzyme treated polyp in DMEM/F-12 media (Dulbecco’s Modified Eagle’s Medium, Ham’s F-12 (1:1 mix), Lonza, USA). By immunostaining against cytokeratin, detached cells were confirmed as epithelial cells. Then, detached epithelial cells were cultured or used for extraction of RNA and protein.

For extraction of total RNA, the tissue or detached epithelial cells were resuspended with 1 ml of QIAzol lysis reagent (Qiagen, USA) and incubated for 5 minutes at room temperature. Cell suspensions were then mixed with 200 μl of chloroform, shaken briefly, and incubated for 3 minutes at room temperature. After centrifugation, the transparent upper layer containing total RNA was mixed with 500 μl of isopropanol, and incubated for 5 minutes on ice. After centrifugation, precipitates were washed with 1 ml of 75% ethanol in DEPC water and centrifuged again. The resulting precipitates were then dried and resuspended with DEPC water. cDNA was synthesized from RNA using a cDNA synthesis master mix (GenDEPOT, USA).

For extraction of protein, detached epithelial cells were resuspended in 200 μl of 5X SDS sample buffer and boiled for 10 minutes at 100°C.

### Epithelial cell culture

To investigate the effect of blockage of E-cadherin or the responsiveness to cytokines of NP epithelial cells, detached epithelial cells from a normal ethmoid and NP were harvested and cultured. Epithelial cells were resuspended in bronchial epithelial cell growth medium (BEGM, containing bovine pituitary extract, insulin, HC, GA-1000, retinoic acid, transferrin, triiodothyronine, epinephrine and hEGF, Lonza, USA) and cultured at 37°C in a 5% CO_2_ atmosphere. Upon reaching 80% confluence, cells were trypsinized, neutralized by trypsin neutralizing solution (Lonza, USA), and subcultured in BEGM. Upon reaching confluency, the subcultured epithelial cells from normal ethmoid were incubated with 1 or 5 μg/ml of anti-E-cadherin neutralizing antibody (DECMA, Santa Cruz Biotechnology, USA) and the subcultured epithelial cells were treated with either 100ng/ml of Th1 cytokines, TNF-α and IFN-γ, or of Th2 cytokines, IL-4 and IL-5 (PeproTech, USA). After overnight incubation, protein or RNA was extracted from the control and treated cells as detailed above.

### Real-time PCR

Gene expression in the whole tissues or epithelial cells was measured by quantitative real-time PCR. The prepared cDNA was amplified and quantified using SYBR green master mix (Qiagen, USA) with the following primers: E-cadherin, forward 5’-TGC TCT TGC TGT TTC TTC GG-3’ and reverse 5’-TGC CCC ATT CGT TCA AGT AG-3’; CCL17, forward 5’-TCC CCT TAG AAA GCT GAA GAC-3’ and reverse 5’-ACT GCA TTC TTC ACT CTC TTG-3’; CCL22, forward 5’-TTG GAG GAG GCC ATT TCA C-3’ and reverse 5’-CAC TCC CCA CAC TTT CAA C-3’ and GAPDH, forward 5’-GAG TCA ACG GAT TTG GTC GT-3’ and reverse 5’-TTG ATT TTG GAG GGA TCT CG-3’. PCRs were performed using a real-time thermal cycler system (TP800/TP860, Takara, Japan) with 40 cycles of a two-step reaction consisting of denaturation at 95°C for 15s followed by annealing/extension at 60°C for 45s. Data was analyzed using the Δ*Ct* method.

### Immunohistochemistry

To evaluate the distribution of E-cadherin and CCL17, tissues were paraffinized and sectioned for immunohistochemistry. In brief, after deparaffinization and hydration, sections were incubated with citrate buffer for antigen retrieval and then treated with H_2_O_2_ to quench endogenous peroxidase activity. After blocking nonspecific areas of sections, each section was incubated with anti-E-cadherin antibody (Santa Cruz Biotechnology, USA) or anti-CCL17 antibody (Thermo Scientific, USA) followed by the appropriate biotinylated secondary antibody. Antigen-antibody complexes were detected using an avidin-biotin complex detection system (Vectastain ABC Kit, Vector Laboratories, Burlingame, CA, USA). Sections were stained with a DAB Substrate kit (Vector Laboratories), and then briefly counterstained with hematoxylin. The sections were examined with an Olympus BX51 microscope (Tokyo, Japan). Pictures were captured using an Olympus DP72 with DP2-BSW software.

### Western blotting

Protein extracts from epithelial cells were used for Western blotting. Briefly, equal amounts of total protein were separated by 8% SDS-polyacrylamide gel electrophoresis and transferred to nitrocellulose membranes. Membranes were blocked with TBS-Tween-20 (TBS-T) containing 5% skim milk and incubated overnight at 4°C with anti-E-cadherin, anti-CCL17, anti-EGFR, or anti-β-actin antibody (Santa Cruz Biotechnology, USA). Next, membranes were incubated with the appropriate anti-rabbit or anti-mouse antibody (Santa Cruz Biotechnology, USA). Antibody reactions were detected using an ECL detection kit, and proteins were visualized with a ChemiDoc imaging system (Bio-Rad, Hercules, CA, USA).

### Statistical analysis

Statistical analyses were carried out using SPSS for Windows (version 16.0.0; SPSS, Chicago, IL, USA). Data in graphs are expressed as the mean±SE of at least three independent determinations. Differences between the normal ethmoid and polyp samples were determined using a Student’s *t*-test and differences between cytokine treated groups were calculated using one-way ANOVA. p-values <0.05 were considered significant.

## Results

### CCL17 expression was decreased in NP epithelium

**Fig 1:**
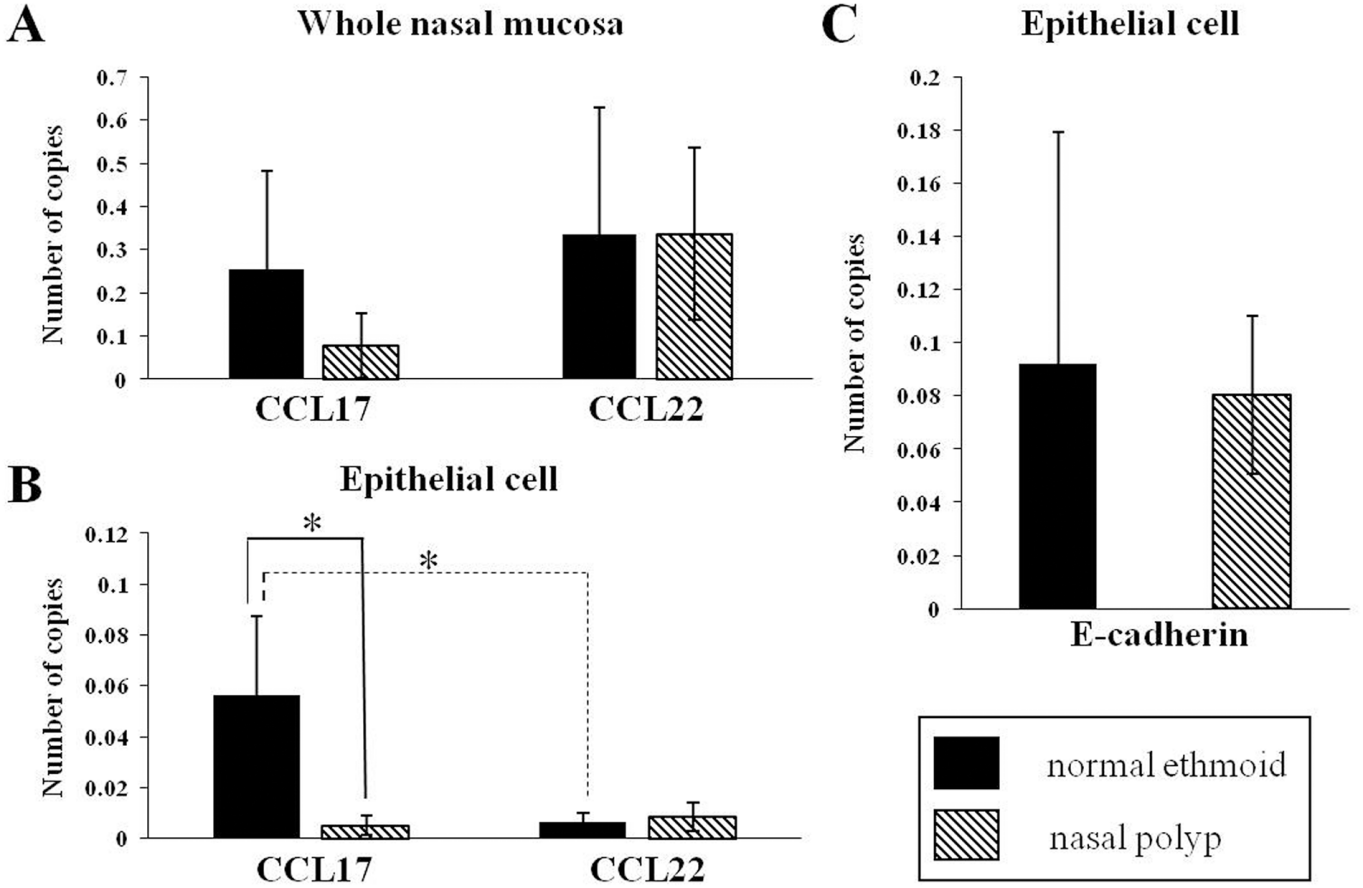
Transcriptional analysis of CC chemokines and E-cadherin in normal ethmoid and nasal polyp cells. CCL17 mRNA and CCL22 mRNA were measured in whole nasal mucosa (A) and nasal mucosa epithelial cells (B). E-cadherin mRNA in nasal mucosa epithelial cells (C) was analyzed. * *p*<0.05.

**Fig 2:**
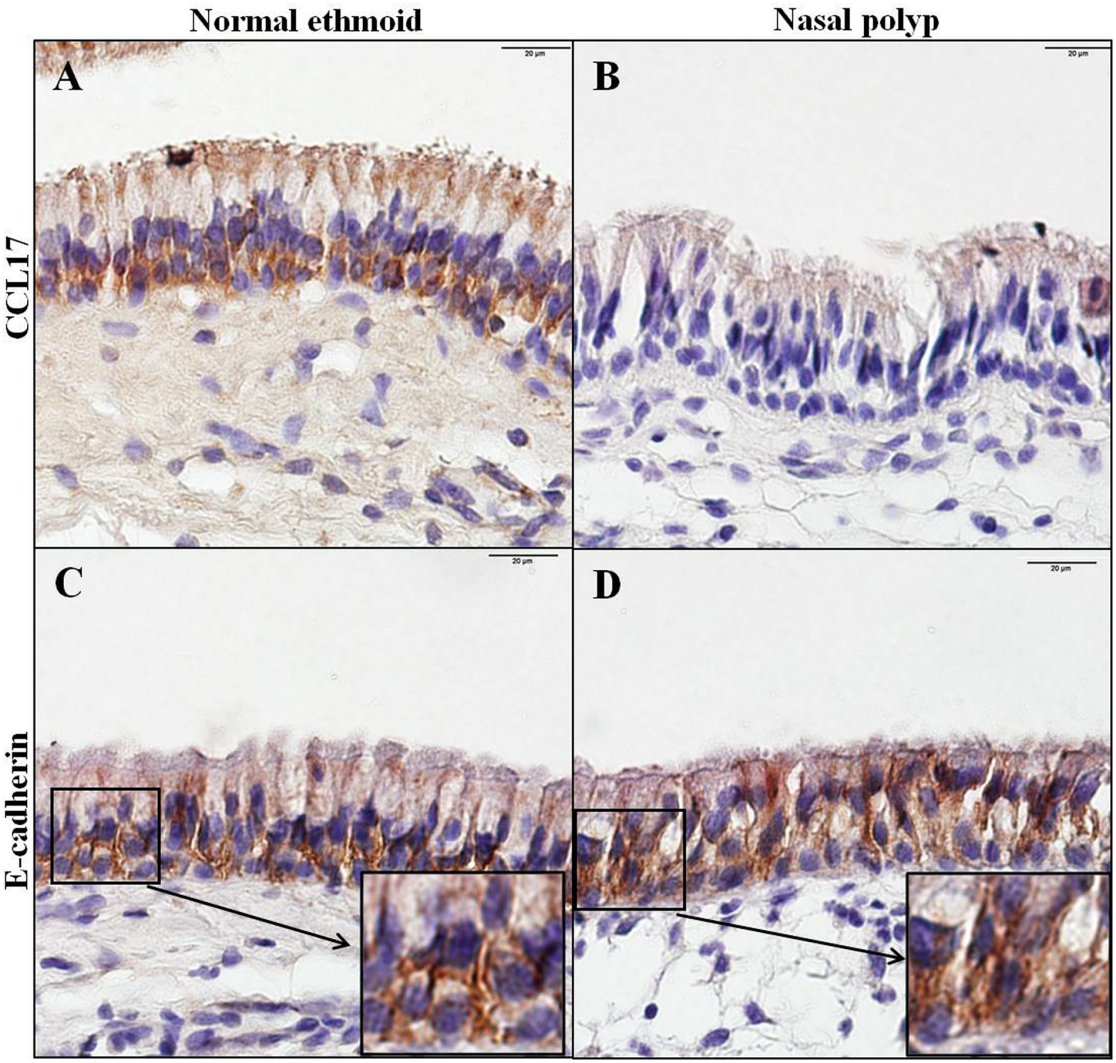
Immunohistochemical analysis of CCL17 and E-cadherin in normal ethmoid and nasal polyp cells. Normal ethmoid (A and C) and nasal polyp (B and D) cells were stained with an anti-CCL17 antibody (A and B) or anti-E-cadherin antibody (C and D). Insets in C and D show the distributional difference of E-cadherin in the normal ethmoid and nasal polyp epithelium. Bar = 20μm.

To examine the expression of Th2 cell recruiting chemokines in the CRSwNP, CCL17 and CCL22 mRNA were measured by real time PCR. In the whole tissue homogenate, the transcriptional expression of CCL17 in the NP was less, even though not statistically different, than that in normal ethmoid sinus mucosa whereas the mRNA level of CCL22 was not different between the normal ethmoid sinus mucosa and NP (Fig 1A). When the mRNA expression of both chemokines was evaluated in the nasal epithelium, CCL17 expression in the normal ethmoid sinus was greater than that in the NP, and the difference between the two tissues was greater than in the tissue homogenate. The expression of CCL22 mRNA in the NP epithelium was similar to that of the normal ethmoid sinus epithelium, as with the tissue homogenates. Intriguingly, the expression of CCL22 mRNA was much less than the transcriptional level of CCL17 in the nasal epithelium, suggesting that CCL17, not CCL22, is mainly expressed in the nasal epithelium (Fig 1B). Reduced expression of CCL17 in the polyp epithelium was also demonstrated by immunohistochemical evaluation (Fig 2A and 2B).

### Disruption of E-cadherin was inversely related to the expression of CCL17 in NPs

**Fig 3:**
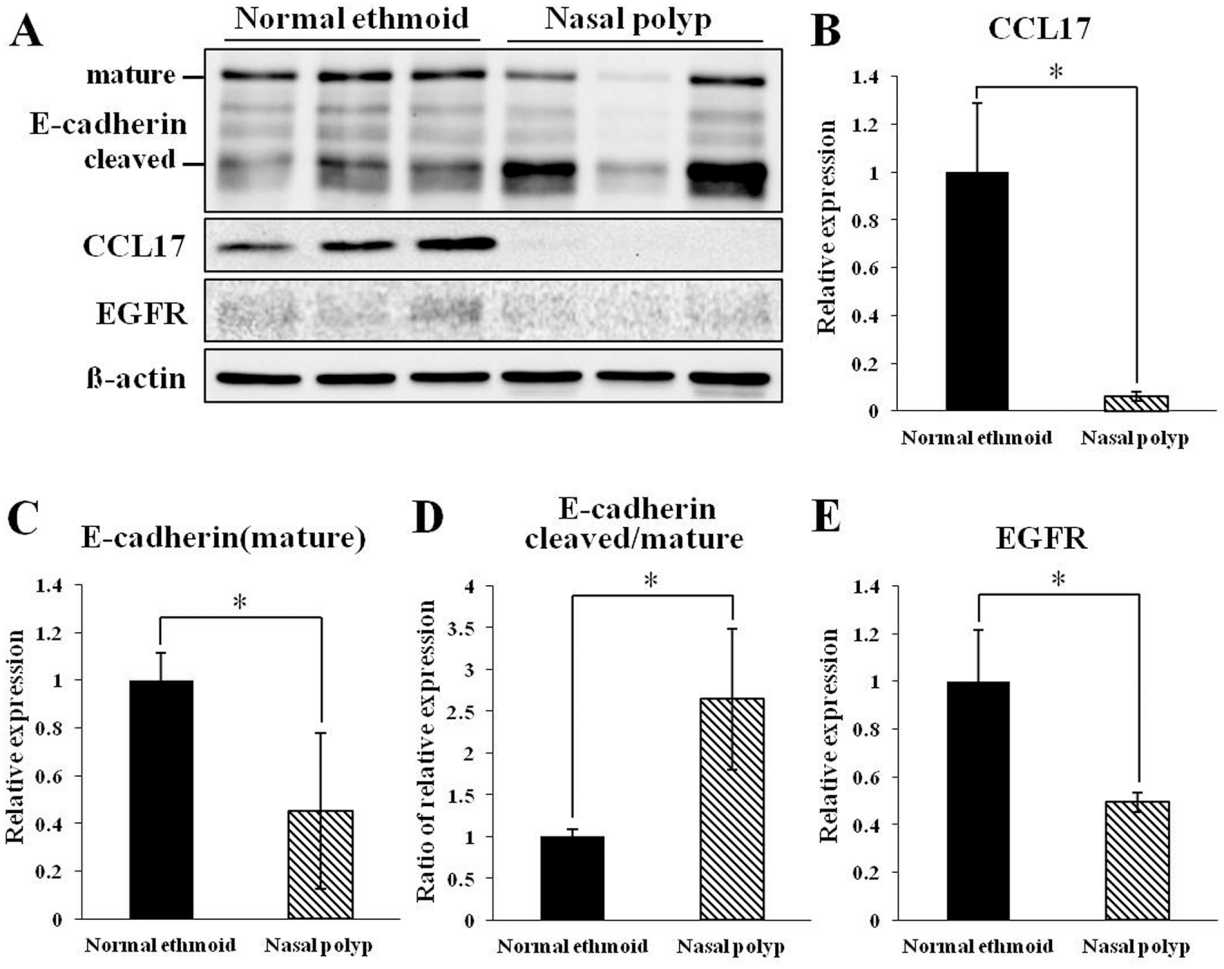
Immunoblotting analysis of E-cadherin, CCL17, and EGFR in epithelial cells from normal ethmoid and nasal polyp cells. Representative immunoblot images (A) and graphical and statistical analysis of relative CCL17 expression (B), relative mature E-cadherin expression (C), the ratio of cleaved E-cadherin to mature E-cadherin (D), and relative EGFR expression (E). * *p*<0.05.

To investigate the relationship between E-cadherin and CCL17 in NPs, transcriptional expression of E-cadherin was measured in the nasal epithelium. Unexpectedly, the mRNA level of E-cadherin was similar in both the normal ethmoid sinus mucosa and NP (Fig 1C). However, when the distribution of E-cadherin was observed by immunostaining, E-cadherin in the normal epithelium was located primarily in the junction of adjacent cells whereas E-cadherin in the NP epithelium was detected in both the cytosol and the junction (Fig 2C and 2D), suggesting that disruption of E-cadherin binding occurred in the NP epithelium. This suggestion was confirmed by immunoblotting of the epithelial cell extract against E-cadherin. The mature E-cadherin expression in the NP epithelial cells was less than in the normal ethmoid sinus mucosa and the ratio of cleaved E-cadherin over mature E-cadherin was increased in the NP epithelial cells (Fig 3A, 3C and 3D). In addition, the protein expression of CCL17 was greatly reduced in the NP epithelial cells, shown by both transcriptional and immunohistochemical examination (Fig 3A and 3B). Interestingly, EGFR expression was also less in the NP epithelial cells compared to the normal ethmoid sinus epithelial cells (Fig 3A and 3E), suggesting that CCL17 expression induced by EGFR is restricted in CRSwNPs.

### Regulation of CCL17 expression by E-cadherin and Th2 cytokines

**Fig 4:**
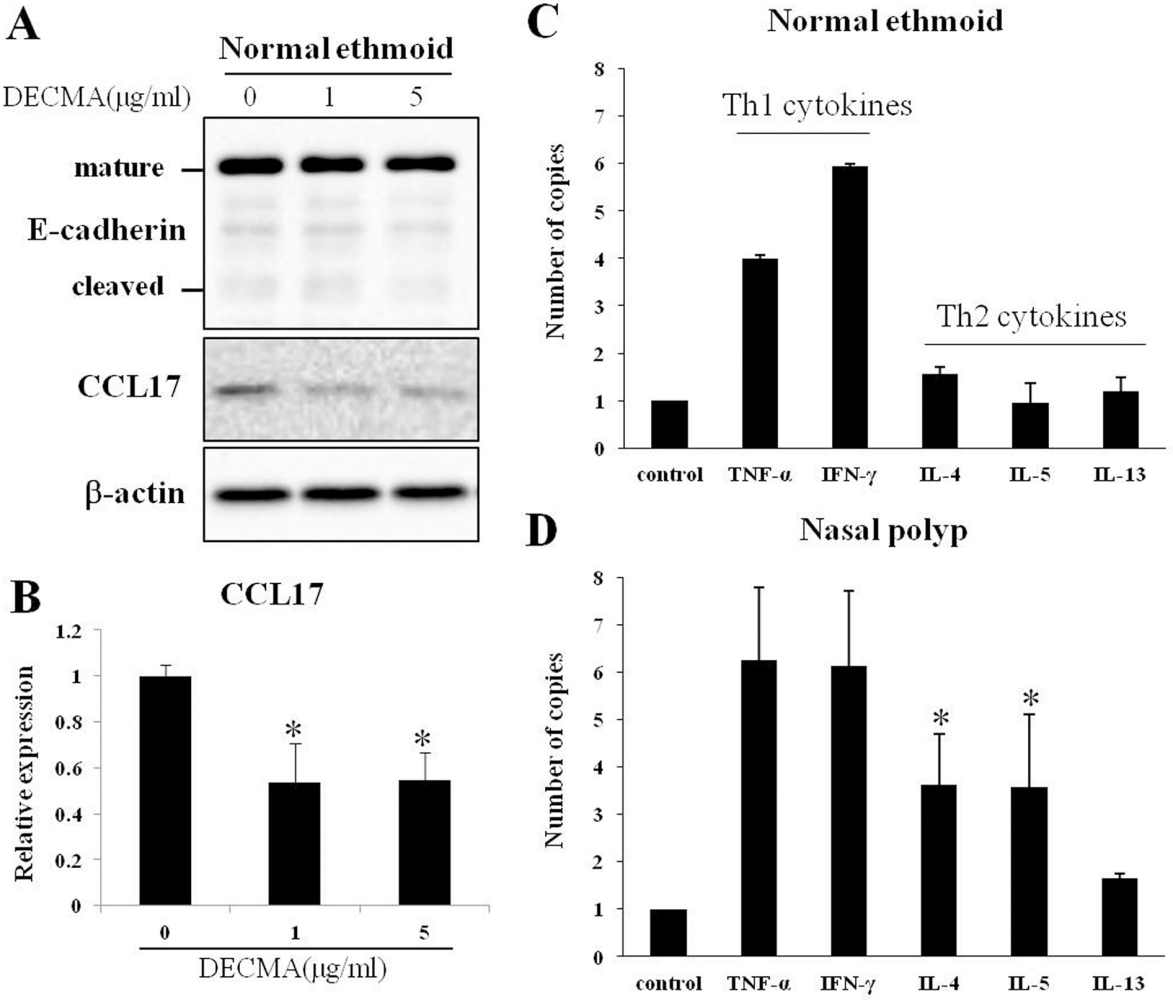
Analysis of CCL17 expression in epithelial cells treated with DECMA or cytokines. Epithelial cells from the normal ethmoid were incubated with DECMA, anti-E-cadherin antibody, and the cell extract was analysed by immunoblotting with anti-E-cadherin and anti-CCL17 antibody. Representative immunoblot images (A) and graphical and statistical analysis of relative CCL17 expression (B). Epithelial cells from the normal ethmoid (C) and nasal polyp (D) were treated with Th1 cytokines TNF-α and IFN-γ or Th2 cytokines IL-4, IL-5 and IL-13 as indicated. The expression of IL-4 and IL-5 in nasal polyp epithelial cells was significantly greater than that of normal ethmoid epithelial cells. * *p*<0.05.

To investigate the correlation between E-cadherin and CCL17, primary epithelial cells from the normal ethmoid sinus were cultured and incubated with anti-E-cadherin neutralizing antibody, DECMA. The expression of CCL17 was decreased as DECMA was added (Fig 4A and 4B), showing that the blockage of E-cadherin in adherence junction reduce the expression of CCL17. Intriguingly, however, the expression of E-cadherin was not decreased because only the binding of E-cadherin was inhibited by DECMA (Fig 4A).

To evaluate the effect of cytokines on CCL17 expression in nasal epithelial cells, primary epithelial cells from the normal ethmoid sinus and NP were cultured and treated with Th1 cytokines or Th2 cytokines. The expression of CCL17 was increased when both epithelial cells were treated with Th1 cytokines, with no statistically significant different between the normal and NP epithelial cells. Intriguingly, when the cells were treated with Th2 cytokines, CCL17 expression increased only in the NP epithelial cells (Fig 4C and 4D). IL-4 and IL-5 significantly increased the expression of CCL17 in NP epithelial cells compared to normal ethmoid sinus cells (p < 0.05). However, after treatment with the other Th2 cytokine, IL-13, CCL17 expression was greater in the NP cells than in the normal ethmoid sinus epithelial cells, although this was not statistically significant. Taken together, these results indicate that NP epithelial cells are more responsive to Th2 cytokines than normal ethmoid sinus epithelial cells.

## Discussion

The results of the present study reveal that the expression of CCL17 is lower in the NP epithelium compared with the epithelium of normal ethmoid sinus mucosa. With respect to the epithelial barrier, E-cadherin in the normal epithelium was located typically at the junction of adjacent cells whereas E-cadherin in the NP epithelium was detected in the cytosol, suggesting that the disruption of E-cadherin binding occurred in the NP epithelium and the expression of cleaved E-cadherin was inversely related to the expression of CCL17 in the NP epithelium. Interestingly, stimulation of cultured NP epithelial cells with IL-4 and IL-5 resulted in the upregulation of CCL17 expression, while CCL17 expression in cultured epithelial cells of control patients was not affected by stimulation with the same panel of cytokines. Taken together, these results indicate that CCL17 and E-cadherin are constitutively expressed in the epithelium of healthy human ethmoid sinus mucosa and downregulated in NP epithelium, suggesting that CCL17 expression may be altered in response to a defective epithelial barrier and/or vulnerability to Th2 cytokines, possibly alluding to their importance in the pathophysiology of NP. This is the first detailed analysis comparing the expression, localization, and regulation of CCL17 and E-cadherin in the NP and healthy ethmoid sinus mucosa epithelium.

Due to the heterogeneity, NPs are divided into two subtypes such as eosinophilic and non-eosinophilic NPs according to the ratio of infiltrating eosinophil number to total inflammatory cells number [24, 25]. From an immunologic perspective, eosinophilic NP is associated with a Th2 polarization, whereas non-eosinophilic NP is associated with the combination of Th1 and Th2 cell polarization [24, 26]. Moreover, Bachert C. reported the majority of NPs from Asian population showed a non-eosinophilic NP [27]. This result is in close agreement with that of the present study showing the majority of NPs is non-eosinophilic NP (Table I). However, to evaluate the possible difference between eosinophilic NP and non-eosinophilic NP, further studies using eosinophilic NPs are required.

Airway epithelium plays a role as a primary protective barrier against inhaled pathogens, allergens, and other irritants. In addition to its passive barrier function, airway epithelial cells actively modulate the immune system to maintain the epithelial integrity. Thus, disintegration of the airway epithelium with disrupted cell junctions due to severe and chronic stimulation might induce abnormal immune responses, resulting in a disease state. For example, the epithelial barrier in CRSwNP is known to be disrupted. The immunostaining of E-cadherin was decreased in the NP epithelium compared to normal nasal mucosa.[28] However, in our results, E-cadherin immunostaining in polyp epithelium was not quantitatively different from that of the epithelium of normal ethmoid sinus cells, but did have a different distribution. The increased expression of E-cadherin in the cytosol can be explained by the result that cleaved E-cadherin was increased in the polyp epithelium (Fig 3A) and is supported by the previous papers that showed E-cadherin redistribution to the cytosol of murine asthma model epithelium[29] and of human bronchial epithelium stimulated by house dust mites.[30] The disruption of E-cadherin activates EGFR-related signaling through dissociation of the complex formation between E-cadherin and EGFR.[31] In *in vitro* research on the airway epithelium, the phosphorylation of EGFR was enhanced when E-cadherin was downregulated with siRNA[18] or the bronchial epithelial cells were stimulated by an allergen.[32] In the sinus mucosa of CRSwNP patients EGFR immunostaining was shown to be increased.[33] However, EGFR expression in the polyp of CRS was reported differently. In the half of polyps in CRS patients EGFR expression was absent but not in the other half.[34] Interestingly, our result revealed that EGFR expression in the polyp epithelium was decreased, which is supported by the previous report that EGFR expression and phosphorylation in human bronchial epithelial cells are downregulated by prolonged exposure to human dust mites mimicking chronic allergic inflammation.[35]

CCL17 in airway epithelial cells of allergic-type asthma is an important mediator of Th2 cell recruitment through its interaction with CCR4 on Th2 cells.[6] Thus, allergens induce CCL17 expression in airway epithelial cells[32] and Th2 cytokines and TNF-α enhance CCL17 production in human nasal mucosa with allergic rhinitis.[19] However, the basal level of CCL17 amount between normal and allergic epithelial cells was not different significantly.[19] Although the transcriptional level of CCL17 was increased in the whole nasal polyp, this increase of CCL17 with upregulation of TSLP was investigated in eosinophilic nasal polyp.[36] Contrary to the previous paper, CCL17 expression of NP in our study was decreased. This result reminded us that majority of NPs in our study were non-eosinophilic.

In our research, the expression of CCL17 in the NP epithelial cells was reduced with the diminished E-cadherin in the adherence junctions and the decreased EGFR production. In epithelial cell culture, furthermore, the blockage of E-cadherin binding in the adherence junctions reduced the CCL17 expression. This result is contradictory to previous research that the blockage of E-cadherin expression induced EGFR-dependent expression of CCL17 in human bronchial epithelial cells.[18] This inconsistency might be caused by the difference in E-cadherin inhibition. The expression of E-cadherin in our report was reduced only in the adherence junctions whereas the expression in previous paper was totally reduced by inhibition of E-cadherin with siRNA. This discrepancy may also stem from the regulation of regulatory T cells (Tregs) by CCL17 in chronic inflammation. In addition to the mediating action of CCL17 for Th2 immunity, CCL17 also induces Treg migration by interacting with chemokine receptor CCR4 on Treg cells.[15] In CRSwNP, the migration potential of Tregs toward Th2-skewed airway inflammation is decreased and thus Tregs were diminished in tissues and peripheral bloods.[37, 38] The reduced Foxp3+ Tregs is recovered after treatment with momentasone, a steroid for nasal inflammation.[39] Furthermore, when prednisone was treated orally in CRSwNP patients, Tregs and CCR4 were increased and migration of Tregs toward NPs was decreased by treatment of CCR4 antagonist on Tregs.[40] Interestingly, by administration of predisone, the expression of CCL17 in NPs was increased whereas the expression of CCL22 was decreased. Thus, our result of diminished CCL17 in NP epithelial cells might be related to the suppressive role of Tregs on hyperactive immune responses.[41] However, further study will be needed to elucidate the interaction between CCL17 and Tregs.

The mechanism that regulates CCL17 in CRSwNP remains unknown. One possible mechanism is that the elevated levels of cytokines in CRSwNP may result in cytokine-induced regulation of CCL17. *In vitro*, combined stimulation with IL-4 and LPS induced CCL17 production in nasal fibroblasts, but not in lung fibroblast.[42] In 16HBE14o- human bronchial epithelial cells, IL-4 and TGF-β cooperatively enhanced *Der p*–induced CCL17 expression.[32] The amounts of CCL17 production induced by the combined stimulation with TNF-α and IL-4 in human nasal epithelial cells obtained from allergic patients was significantly higher than the CCL17 production in those obtained from control.[19]

In the present study, there was no change in the expression of CCL17 after stimulation of normal epithelial cells with IL-4, 5, and IL-13. However, in the NP epithelial cells these Th2 cytokines increased the expression of CCL17 at the mRNA level. Therefore, this data suggests that NP epithelial cells are more vulnerable to stimulation by Th2 cytokines compared to normal epithelial cells. Taken together, the debating results concerning the effects of cytokines on CCL17 expression may reveal differences in tissue conditions between the diverse investigations in the literature. Further studies to elucidate the molecular mechanisms influencing CCL17 expression are needed to evaluate the specific effects of cytokines in NP epithelial cells.

## Conclusions

This is the first report investigating the association between the epithelial barrier and CCL17 expression in CRSwNP. Our results suggest that an altered expression of CCL17 in defective NP epithelium may be involved in the pathogenesis of NP and can be differentially modulated by cytokines in the nasal epithelium of patients with CRSwNP compared with healthy individuals. Further studies are needed to evaluate the functional role of CCL17 in the pathogenesis of NP with *in vitro* analyses.
